# Deep Brain Stimulation Surgery Using a Mobile Intraoperative CT Scanner

**DOI:** 10.7759/cureus.29139

**Published:** 2022-09-13

**Authors:** Daniel Cavalcante, Muhammad S Ghauri, Ryder Gwinn

**Affiliations:** 1 Neurosurgery, University of Colorado Anschutz Medical Campus, Aurora, USA; 2 Neurosurgery, California University of Science and Medicine, Colton, USA; 3 Neurological and Spine Surgery, Swedish Neuroscience Institute, Seattle, USA

**Keywords:** euclidean distance, movement disorders, leksell localizer box, intraoperative ct scanner, deep brain stimulation (dbs)

## Abstract

Introduction

Deep brain stimulation (DBS) is widely used for the treatment of movement disorders. Precise placement of electrodes is critical for treatment success. The aim of this study was to analyze the accuracy of the intraoperative computer tomography (CT) images compared to that of a traditional fixed CT for patients undergoing DBS procedures.

Methods

We retrospectively analyzed the charts from 30 patients who underwent DBS. In group 1, 10 patients underwent electrode implantation surgery using a fixed CT scanner for pre- and post-operative (OP) images. In group 2, 20 patients underwent surgery using an intraoperative CT scanner for pre- and post-operative images, as well as a fixed CT scanner for post-operative images. We compared the average pre-operative localizer box registration error acquired in these two groups. We also analyzed, in group 2, the final electrode position given on each post-operative CT images. We compared the average Euclidean distances between each set of cartesian coordinates to assess target accuracy between both scanning methodologies.

Results

Thirty patients had ages ranging from 40 to 88 years, with a median of 69 years old. In the 20 patients who utilized an intraoperative CT scanner pre-operatively in group 2, the mean error, given by the Medtronic software (Medtronic Minimally Invasive Therapies, Minneapolis, MN) with the Leksell frame on, was 0.37. For the 10 pre-operative scans with the stealth fixed CT scanner in group 1, the mean error was 0.44 (p = 0.13). In group 2, the average of the 20 Euclidean distances for each target, in those 20 patients who had post-operative images with both scanners, was 0.36.

Conclusion

We concluded that the accuracy of the intraoperative CT scanner is comparable to the gold standard fixed CT scanner for DBS electrode planning and placement, as well as for positioning confirmation after the electrodes are in place.

## Introduction

Deep brain stimulation (DBS) has been approved by the Food and Drug Administration (FDA) for the treatment of movement disorders such as Parkinson’s disease, essential tremor, and dystonia since 1997. Recent studies have investigated the utility of DBS in psychiatric conditions such as depression, obsessive-compulsive disorder, and Tourette’s. Precise placement of the electrode is critical for treatment success [1,2], and high-quality imaging studies are critical for optimal target planning.

Typically, frame-based techniques for electrode placement utilize a pre-operative (OP) MRI scan for target planning, as well as a computer tomography (CT) scan after the frame and localizing box have been placed on the patient. A new generation of mobile scanners now allows for CT scans to be performed in the operating room (OR), potentially allowing for more efficient workflow and intraoperative confirmation of hardware placement for cranial [3] and spine procedures [4-6]. Few studies have compared the accuracy of various intraoperative CT scanners (e.g., O-arm {Medtronic Minimally Invasive Therapies, Minneapolis, MN}) with post-operative MRI and fixed CT scans [7-17]. More studies are needed to explore the performance and utility of intraoperative scanners in DBS surgery.

Our goal was to assess the real-world accuracy of the intraoperative CT scanner (AIRO, Brainlab, Munich, Germany) to that of a fixed CT (Siemens, Berlin, Germany) for patients undergoing DBS procedures. Our first measurement is the localizer headbox registration error generated by the Framelink software version 5.4 (Medtronic Minimally Invasive Therapies, Minneapolis, MN). This error is larger when the CT scan of the localizer box is less precise. Scan precision may be affected by patient movement, scanner movement, localizer box warping, field distortion, or inter-slice distance variability during scan acquisition [15,16]. A more accurate scan with fewer of these variables will produce a smaller registration error. Regardless of the source, this error will ultimately be a determining factor in the accuracy of lead placement and should therefore be minimized. Our second measurement will be the final electrode position given by both intraoperative and the gold standard fixed CT scanner. We hypothesized that utilizing the intraoperative CT scanner would produce a comparable registration error and similar final electrode position to the traditional fixed CT scanner.

## Materials and methods

Software analysis

First, we utilized the automatic detection of the Leksell frame localizer box fiducials built into the Framelink software version 5.4 (Medtronic Minimally Invasive Therapies, Minneapolis, MN). This software automatically localizes the nine copper fiducials embedded in the Leksell localizer box for each slice of the acquired scan. Next, it determines how close the observed locations of the fiducials are to the expected location in the idealized model. It then calculates a registration error based on this comparison. We compared the localizer headbox error for scans acquired on our fixed CT scanner in group 1 to the error obtained when scanning patients with our intraoperative CT scanner (AIRO, Brainlab, Munich, Germany) in group 2. Our second assessment, in group 2, compares the final electrode position captured by our intraoperative CT to the position captured by our standard post-operative fine cut CT scan acquired on our fixed CT scanner, after the surgery had been completed. To determine the final electrode position, a single observer measured the electrode tip position (x, y, and z) for each post-OP CT scan image. The same target was measured blindly three times on three different days. Brightness and contrast settings were identical in all scans evaluated to use a consistent Hounsfield unit border for the distal contact. The average of these repeated measures was determined for both post-OP images from intraoperative CT scanner and fixed CT scanner. Next, the Euclidean distance was measured between both averages of the same target. Finally, we also measured the average of the Euclidean distances (Euclidean distance formula: dist((x, y, z), (a, b, c)) = √(x - a)² + (y - b)² + (z - c)²) with standard deviation (SD) to compare the accuracy of the mobile CT scanner with the gold standard fixed CT scanner.

Study design

Thirty patients were divided into two study groups. Group 1 consisted of 10 patients who underwent electrode implantation surgery using a fixed CT scanner to acquire pre- and post-operative images. Group 2 consisted of 20 patients who underwent electrode implantation surgery using an intraoperative CT scanner for pre- and post-operative images. These 20 patients also had post-operative images with the same fixed CT scanner as the first 10 patients for accuracy determination and to assess for post-operative bleeding.

The charts from these 30 patients who received deep brain stimulation treatment from August 1, 2014, to September 30, 2015, were analyzed. There were two primary outcome measures: 1) Head box localizer error: This measurement is given by the Framelink software (Medtronic Minimally Invasive Therapies, Minneapolis, MN) when registering the patient with a Leksell CT localizer. We compared localizer box registration error of group 1 patients who had images acquired outside the operating room, on a fixed CT scanner, with the error found on group 2 patients, who had scans acquired intraoperatively using the mobile intraoperative scanner (AIRO, Brainlab, Munich, Germany). 2) Electrode coordinates for group 2: We utilized the Framelink software to record the Cartesian coordinates of the final electrode position given by both the intraoperative CT and fixed CT scanner. For the group 2 patients, who had two post-operative images with both standard fixed CT images and intraoperative CT images, we determined the X, Y, and Z coordinates of the most distal contact of the electrode in both post-operative image sets.

Statistical analysis

Comparing group 1 and group 2, we performed an independent (unpaired) t-test to evaluate if there were any significant differences in the mean error between both CT methodologies, based on the pre-operative images. In group 2, with the 20 patients who had both post-operative images with the intraoperative CT scanner prior to leaving the OR and post-operative fine cut images on the fixed CT scanner, we utilized the Framelink software to record the Cartesian coordinates (x, y, and z coordinates) of the electrode tip location given by both CT scanners. This allowed us to compare the similarity, in three dimensions of the two imaging techniques. Next, we calculated the Euclidean distance between the average coordinate of the electrode tip given by the intraoperative CT scanner and the fixed CT scanner. The average of our 20 Euclidean distances was 0.36 mm (SD of 0.19).

## Results

Overall, 30 patients were included in this study. Seventeen were male, and 13 were female with ages ranging from 40 to 88 years, with a median of 69 years old. Our electrode positions were targeted to the ventral intermediate thalamus (VIM) in 13 patients, the subthalamic nucleus (STN) in 14 patients, and the globus pallidus internus (GPI) in three patients (Table 1).

**Table 1:** Patient descriptive characteristics VIM: ventral intermediate thalamus; STN: subthalamic nucleus; GPI: globus pallidus internus

Variable	Value (n = 30)
Age	69 ± 14
Sex	
Male	17 (57%)
Female	13 (43%)
Target	
VIM	13 (43%)
STN	14 (47%)
GPI	3 (10%)

**Figure 1:**
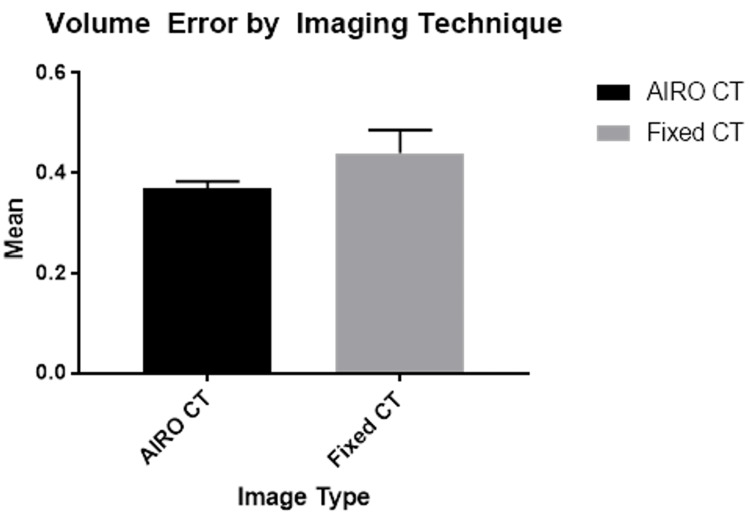
Comparison of Leksell headframe box errors produced in pre-operative scans using intraoperative CT (AIRO) with fixed CT scanner

We measured the pre-operative error given by the Framelink software with the Leksell localizer box (Figure 1). In the 20 patients that had the pre-operative scans with the intraoperative CT scanner, the mean error was 0.37 (SD of 0.01). In the 10 pre-operative scans with the fixed CT scanner, the mean error was 0.44 (SD of 0.05) (P = 0.13) as shown in Table 2.

**Table 2:** Unpaired t-test results between our two study groups SEM: standard error of the mean

Variable	AIRO	Fixed CT
Mean	0.37	0.44
SD	0.013	0.046
SEM	0.030	0.015
P-value	0.126	

**Figure 2:**
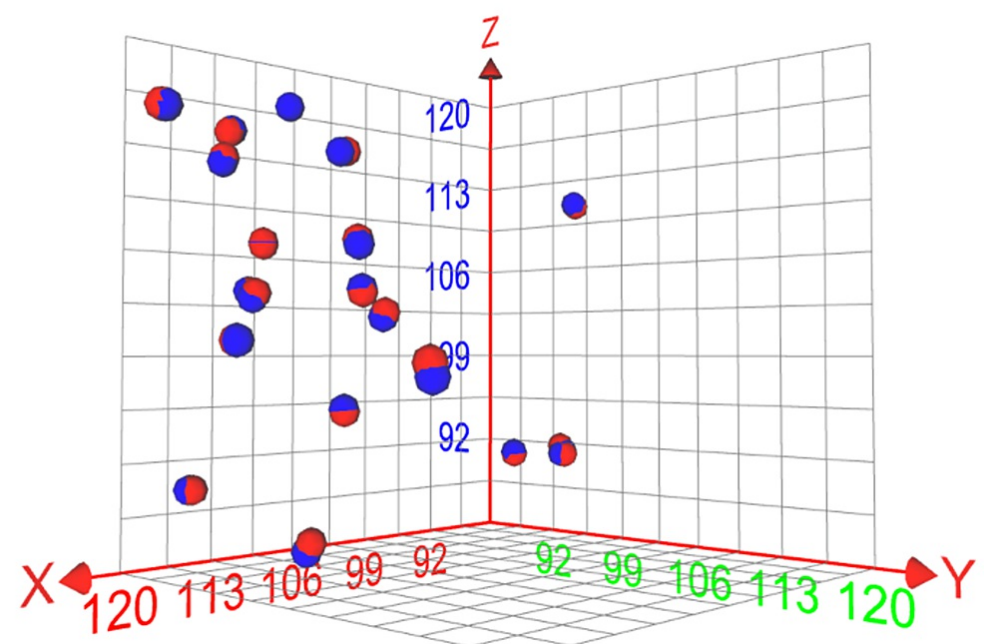
Three-dimensional plot showing the spatial difference between the average X, Y, and Z electrode tip coordinates using intraoperative (AIRO) CT scan (red) and fixed CT scan (blue)

In the 20 patients who had both post-operative fine cut intraoperative CT scans prior to leaving the OR and post-operative fine cut scans on the fixed CT scan, we utilized the Framelink software to record the Cartesian coordinates (x, y, and z coordinates) of each electrode tip. The graphical representation of the proximity of the red (intraoperative CT) and blue (fixed CT) circles depicts how well both scans mirror each other (Figure 2). Overall, we had no post-operative bleeding to report in our study.

## Discussion

Deep brain stimulation has been used for two decades to treat movement disorders that are not medically controlled [6]. Accuracy of target planning and final positioning of the electrode are imperative for effective symptom control [7,8]. Burchiel et al. previously demonstrated that with patients under general anesthesia, placing DBS electrodes using an intraoperative CT scanner is at least comparable to the other methods [4]. Sharma et al. compared the accuracy of the O-arm for DBS electrode implantation with the post-operative CT scan and concluded that DBS leads can be implanted safely and accurately using intraoperative O-arm with a frame base target system [9,10]. Servello et al. conducted a retrospective study with 202 patients comparing the intraoperative CT scan with the post-operative fixed Ct and MRI scans and concluded that the intraoperative CT scan is comparable to the post-operative Ct scan but should not substitute the post-operative MRI scan especially for targets visible in the MRI [11]. On the other hand, Bot et al. compared 52 final DBS lead position with both intraoperative CT scan and post-operative MRI and concluded that both images are equivalent [16]. Servello et al. more recently corroborated these findings in a prospective study with 52 DBS patients and found both imaging devices to be easy to use, precise, and safe [14].

In our study, we aimed to prove that the accuracy of the intraoperative CT scanner is comparable to a fixed CT scanner for the purpose of trajectory planning and confirmation of the final electrode position. When we compared the frame registration errors given by the Framelink software in the pre-operative images between the two scanners, we observed a lower error with the intraoperative scanner, although not statistically significant. This may be attributed to the fact that during image acquisition, this unit moves along self-contained tracts rather than on the operating room floor potentially contributing to a higher level of accuracy. However, this study was unable to clearly delineate which variables are causally related and how they contribute to these findings. Future multi-institutional studies are needed to investigate a larger patient population with varying disease contexts to ensure the reproducibility and generalizability of these findings.

Our analysis of post-operative CT images of the final electrode position between the two different CT image modalities in group 2 (Figure 1) shows the average Euclidean distance between electrode positions on the two different scanners was only 0.36 mm (SD of 0.19). This difference of less than a half millimeter is within the error of our ability to measure and document electrode deviations in electrode position post-operatively.

Our study does have some limitations. Given that this is a single institutional study with a limited sample size, this could explain the inability to find statistically significant differences between intraoperative and fixed CT scanning modalities. However, the primary goal for this study was to show that the intraoperative CT scanner performed similarly to the fixed CT scanner, so we expected to find little to no difference. In addition, although we reported no post-operative bleeding, more research is needed to delineate the detection capabilities of intraoperative CT scanners. Finally, we did not perform any correlational analyses to delineate how our specific electrode placement affected patient outcomes. Future multi-institutional studies with a larger and more varied patient population are needed to validate our findings and determine any correlations with patient outcomes.

Given our inability to distinguish significant differences in localizer box registration errors and the fact that electrode positions were measured within 0.36 mm of each other using the different scanners, we can argue that the use of this intraoperative mobile CT scanner is at least comparable to the fixed CT scanner for target planning, as well as for confirmation of the final electrode position. Our data suggests that one could reliably utilize intraoperative CT images to make decisions regarding sub-optimal electrode positioning prior to leaving the operating room. This potentially increases workflow efficiency, which could lead to improved patient safety and outcomes. Given the lack of observed bleeding in these cases, it is unclear whether intraoperative imaging from the intraoperative scanner would be adequate for the imaging of blood products in the acute surgical setting.

## Conclusions

The utilization of intraoperative imaging for the placement of intracranial electrodes allows for improved workflow efficiency during DBS procedures. The fidelity and accuracy of intraoperative CT scanners have been questioned; however, little data exists outlining how these scanners compare to more standard fixed CT scans in real-world application. The intraoperative CT scanner utilized in this study provided very similar accuracy to our gold standard fixed CT. Further studies using the intraoperative imaging in DBS and other electrode placement procedures are needed to better evaluate the safety and efficacy of this imaging modality.
